# The *Trp73* Mutant Mice: A Ciliopathy Model That Uncouples Ciliogenesis From Planar Cell Polarity

**DOI:** 10.3389/fgene.2019.00154

**Published:** 2019-03-15

**Authors:** Margarita M. Marques, Javier Villoch-Fernandez, Laura Maeso-Alonso, Sandra Fuertes-Alvarez, Maria C. Marin

**Affiliations:** ^1^ Departamento de Producción Animal, Laboratorio de Diferenciación Celular y Diseño de Modelos Celulares, Instituto de Desarrollo Ganadero y Sanidad Animal, Universidad de León, León, Spain; ^2^ Departamento de Biología Molecular, Laboratorio de Diferenciación Celular y Diseño de Modelos Celulares, Instituto de Biomedicina, Universidad de León, León, Spain

**Keywords:** TAp73, DNp73, ependymal cells, ciliogenesis, planar cell polarity, microtubules, actin cytoskeleton, hydrocephalus

## Abstract

p73 transcription factor belongs to one of the most important gene families in vertebrate biology, the p53-family. *Trp73* gene, like the other family members, generates multiple isoforms named TA and DNp73, with different and, sometimes, antagonist functions. Although p73 shares many biological functions with p53, it also plays distinct roles during development. *Trp73* null mice (p73KO from now on) show multiple phenotypes as gastrointestinal and cranial hemorrhages, rhinitis and severe central nervous system defects. Several groups, including ours, have revisited the apparently unrelated phenotypes observed in total p73KO and revealed a novel p73 function in the organization of ciliated epithelia in brain and trachea, but also an essential role as regulator of ependymal planar cell polarity. Unlike p73KO or TAp73KO mice, tumor-prone *Trp53*−/− mice (p53KO) do not present ependymal ciliary or planar cell polarity defects, indicating that regulation of ciliogenesis and PCP is a p73-specific function. Thus, loss of ciliary biogenesis and epithelial organization might be a common underlying cause of the diverse p73KO-phenotypes, highlighting *Trp73* role as an architect of the epithelial tissue. In this review we would like to discuss the data regarding p73 role as regulator of ependymal cell ciliogenesis and PCP, supporting the view of the *Trp73*-mutant mice as a model that uncouples ciliogenesis from PCP and a possible model of human congenital hydrocephalus.

## Introduction

The p53-family is constituted by the transcription factors p53, p63 and p73. The tumor suppressive function of p53 largely resides in its capacity to sense potentially oncogenic and genotoxic stress conditions, and to coordinate a complex set of molecular events leading to growth restraining responses and, ultimately, to senescence and/or apoptosis. Thus, p53 is a central node in the molecular network controlling cell proliferation and death in response to pathological and physiological conditions, a network in which p73 and p63 are also entangled (Pflaum et al., 2014). Emerging evidence reveals the role of the p53-family in other biological processes like stem cell self-renewal, cell metabolism, fertility or inflammation (Gebel et al., 2017; Napoli and Flores, 2017; Nemajerova et al., 2018). Despite its function in the maintenance of genomic integrity, the initial analysis of p53KO mice suggested that there were developmentally normal (Donehower et al., 1992). However, posterior in-depth analysis revealed that p53 was essential for normal development (reviewed by Donehower et al., 1992; Shin et al., 2013; Jain and Barton, 2018). *Trp63* and *Trp73* gene targeting studies showed that both genes are also required during embryogenesis (Mills et al., 1999; Yang et al., 2000; Tomasini et al., 2009; Wang et al., 2017). Thus, the emerging picture is that of an interconnected pathway, in which p63 and p73 share many functional properties with p53 but they also claim distinct and unique biological roles (Van Nostrand et al., 2017; Wang et al., 2017).

Moreover, *Trp73* generates functionally different TA and DNp73 isoforms (Candi et al., 2014). Dependent upon the presence of the N-terminal transcriptionally active TA-domain, TAp73 variants exhibit p53-like transcriptional activities and tumor suppressive functions. Conversely, N-terminally truncated DN-isoforms act as dominant-negative inhibitor of p53 and TAp73 and thus, have oncogenic properties (Engelmann and Pützer, 2014). It is noteworthy that the p53 family members not only induce several common target-genes (Grob et al., 2001) but can also regulate each other’s expression and function (Fatt et al., 2014). In agreement with all these complex interactions, compensatory mechanisms in the p53 family knockout models have been reported. Examples of this are the elevated levels of DNp73 mRNA in the constitutive TAp73KO and p63KO mice (Tomasini et al., 2008; Cancino et al., 2015).

## *Trp73* and its Role in CNS Development

p73 fundamental role in brain development and homeostasis was highlighted by the central nervous system (CNS) defects in the p73KO mice. These included third ventricle enlargement, congenital hydrocephalus, hippocampal dysgenesis with abnormalities in the CA1–CA3 pyramidal cell layers and the dentate gyrus, loss of Cajal–Retzius neurons and abnormalities of the pheromone sensory pathway (Killick et al., 2011).

Identification of the predominant p73-isoform in the brain has proven complicated and the compiled data suggest a differential regulation of p73-isoforms expression during development that varies among cell types. Initial studies indicated that DNp73-isoforms were the predominant isoforms detected in sympathetic neurons *in vivo*, where they act as a survival factor (Pozniak et al., 2000, 2002; Yang et al., 2000); they were also expressed in Cajal–Retzius cells and in the choroid plexus (Meyer et al., 2004; Tissir et al., 2009; Hernández-Acosta et al., 2011). The analysis of DNp73-specific KO mice (DNp73KO) (Tissir et al., 2009; Wilhelm et al., 2010) confirmed the neuro-protective role of DNp73 and attributed most of the neurological phenotypes to its loss. However, DNp73KO mice did not develop striking hippocampal abnormalities like the p73KO, nor hydrocephalus, indicating that TAp73-isoforms may contribute to the development of the CNS. Nevertheless, in one of the DNp73KO models (Wilhelm et al., 2010), mutant mice had enlarged ventricles (Killick et al., 2011), suggesting a possible role of DNp73.

Expression of TAp73 appears to be restricted to specific brain areas. TAp73 was detected in the cortical hem, where it has a role in brain cortical patterning and in mature ependymal cells (ECs) of wild-type ventricles, which strongly express p73 (Medina-Bolívar et al., 2014; Fujitani et al., 2017). DNp73 has not been detected in these cells or in in its precursors, the radial glial cells (RGC) (Tissir et al., 2009). In addition, TAp73 is the predominant isoform expressed in embryonic neural stem cells (NSCs) (Tissir et al., 2009; Agostini et al., 2010; Medina-Bolívar et al., 2014) and has been shown to regulate NSCs stemness and brain neurogenic niche maintenance and cytoarchitecture (Agostini et al., 2010; Fujitani et al., 2010; Gonzalez-Cano et al., 2010, 2016; Talos et al., 2010). In agreement, TAp73KO mice show hippocampal dysgenesis with loss of the lower blade of the dentate gyrus like that in p73KO, but do not have ventricle enlargement or hydrocephalus (Tomasini et al., 2008), altogether posing the possibility of compensatory mechanisms in the absence of one of the isoforms.

## *Trp73* is Required for Radial Glial to Ependymal Cell Fate Determination

**Figure 1:**
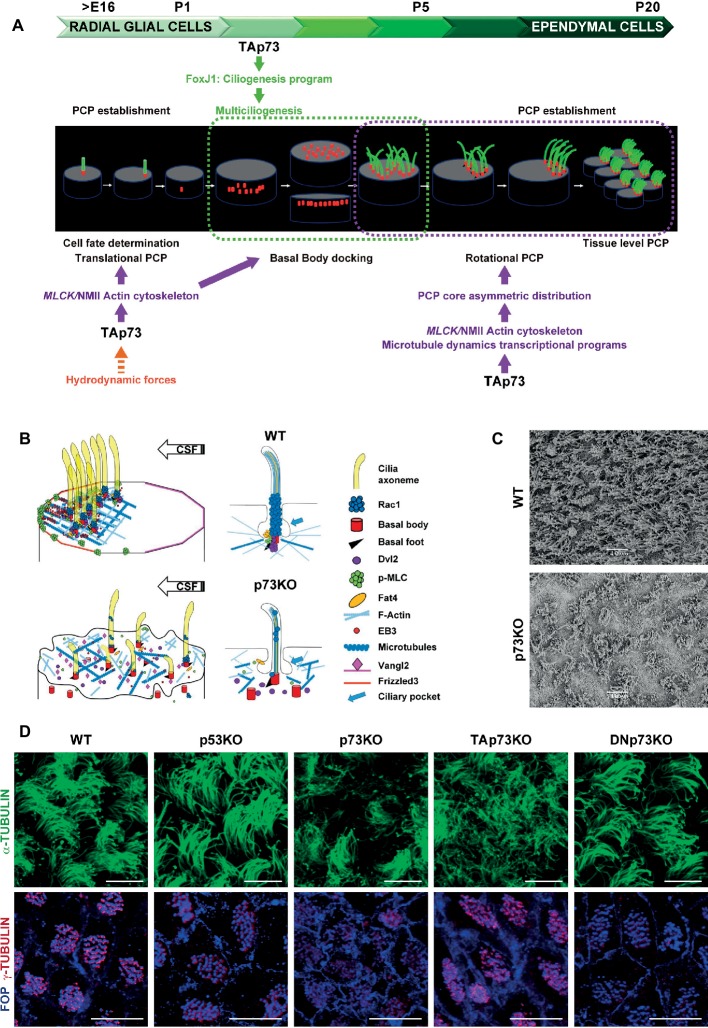
*Trp73* deficiency affects ciliogenesis and the planar polarization of microtubule and actin networks resulting in lack of PCP and cilia disarrangement in ependymal cells. **(A)** Schematic representation of TAp73 regulation of PCP and ciliogenesis during the development from RGCs to ECs cells in rodent brain ventricular epithelia. Translational PCP begins by embryonic day E16, when RGCs primary cilium is displaced towards the anterior apical surface instructed by mechanosensory signaling. At this stage, TAp73 regulation of NMII activation is required for proper actin cytoskeleton dynamics, essential for tPCP establishment. Multiciliogenesis starts postnatally (P2) during the initial steps of ECs differentiation. TAp73 plays a central role activating transcriptional multiciliogenesis programs, but it is possible that p73-regulation of actin networks impinge on basal body (BB) docking. Around P5 motile cilia of immature ECs are randomly distributed on the apical surface. TAp73 regulation of polarized microtubules dynamics is important for the asymmetric localization of PCP-core proteins at opposite sides of the apical membrane at MT anchoring points at cell junctions. Asymetric PCP-core complexes instruct BBs to become aligned as the ependymal layer matures and rotational polarity is established with the formation of signaling complexes at the BBs, including Dvl and Rac1. At later stages (P15) TAp73 function is required for the establishment of tPCP in ECs which will be coordinated at tissular level. **(B)** Schematic representation of the subcellular localization of PCP-regulatory proteins and the effects of p73 deficiency in ependymal cells. The polarized distribution of activated NMII puncta at apical cell-junctions, and its association with the BBs (red) of crescents-BB clusters, sustains the establishment of polarized actin lattices (light blue). Activated NMII is depicted by p-MLC (green). Polarized cortical MT networks (dark blue) grew asymmetrically (EB3, red solid circle) from the center of the cell towards the anterior region contacting the plasma membrane at MT-anchoring points. These actin and microtubule networks allow the junctional localization of PCP-core protein complexes [Vangl2 (pink) and Frizzled (orange)] and the formation of signaling complexes at BB-clusters and at the base of cilia axoneme [Dvl2 (purple), Rac1 (blue) and Fat4 (yellow)]. p73 deficiency results in severe defects in ciliogenesis and the loss of apical cytoskeleton dynamics in ECs, which correlates with impaired BB docking, lack of asymmetric localization PCP-proteins and the disassembly of the signaling complexes associated with the cilia BB, resulting in lack of translational and rotational polarity in these cells and disorganization of cilia. **(C)** Representative scanning electron microscopy (SEM) images of WT and p73KO lateral ventricle wall (LW) whole-mounts (WMs) at P15. Scale bar 10 μm. **(D)** Regulation of ciliogenesis and PCP is a p73-specific function. Representative confocal images of WT, p53KO, p73KO, TAp73KO and DNp73KO LWs stained with α-tubulin to visualize the ciliary axoneme, or combined staining of FOP, which localized at the base of cilia, and γ-tubulin for the basal feet, to delineate the orientation of each cilium (rPCP). BBs from WT, p53KO and DNp73KO ECs were aligned in parallel rows that contained similar numbers of regularly spaced BBs, while in p73KO and TAp73KO cells the stereotypic arrangement of cilia (spacing and number of cilia per row) was severely impaired. The figure includes original images that reflect previously demonstrated data (Fuertes-Alvarez et al., 2018). Scale bar 10 μm.

The subventricular zone (SVZ) is one of the prominent regions of neurogenesis in the adult rodent brain and is located along the walls of the lateral ventricles next to the ependyma. The adult SVZ is a highly organized microenvironment comprised by multiciliated ECs that wrap around monociliated NSCs, forming organized neurogenic structures denominated pinwheels, which play a fundamental role in the maintenance of neurogenesis (Mirzadeh et al., 2010). In rodents, the walls of the lateral ventricles at birth maintain many similarities to the ventricular zone of the immature neuroepithelium but will change dramatically during postnatal development. The ECs that layer the wall of the lateral ventricle are derived from monociliated radial glial cells (RGCs) (Figure 1A). During perinatal development, a special subset of RGCs will lose their morphology and will give raise to multiciliated ECs (Spassky et al., 2005; Kriegstein and Alvarez-Buylla, 2009). This transformation requires RGC cell fate determination and EC differentiation (Merkle et al., 2004; Guirao et al., 2010). While specification of RGCs begins around E16, EC differentiation is initiated after birth and completed by P20, following a multistep process that includes multiciliogenesis and PCP (Ohata and Alvarez-Buylla, 2016; Kyrousi et al., 2017) (Figure 1A).

The hydrocephalus phenotype of p73KO suggested a possible p73 role in ependymal ciliary function that might be linked to its regulation of the neurogenic environment. Indeed, RGCs express p73 (Fujitani et al., 2017) and in its absence RGCs transition to ECs is altered generating immature cells with abnormal identities and morphology (Gonzalez-Cano et al., 2016). The specification of RGCs towards NSCs or ECs is identified by either GFAP or *GemC1* and *Mcidas* expression, respectively (Kyrousi et al., 2015). Interestingly, in the SVZ of p73KO brains, aberrant S100β^+^-ECs that also expressed GFAP were detected (Gonzalez-Cano et al., 2016), indicating that lack of p73 results in alterations in cell fate determination transcriptional program of RGCs. Moreover, Fujitani and colleagues proposed that embryonic primary ciliogenesis may be regulated by p73, since they found that disruption of p73 (both TA and DNp73) during early postnatal EC development (P1-P5) did not cause hydrocephalus (Fujitani et al., 2017). The compiled data strongly support the idea that p73 functions at several stages during RGCs transformation into EC (Figure 1A), beginning at an earlier developmental stage before activation of *Mcidas* and *FoxJ1*, master regulatory genes of multiciliogenesis.

p73 function in MCC fate specification in tracheal epithelium is controversial. p73 fate specification function was proposed in a subset of basal cells that co-express p73 and p63, where p73 expression identified them to undergo MCC differentiation (Marshall et al., 2016). However, a second research group, using tracheal epithelium cultures (MTEC), concluded that p73 is downstream of *Mcidas* and functions mainly after MCC fate specification (Nemajerova et al., 2016). Both groups identified over 100 putative p73 target genes that regulate MCC differentiation and homeostasis and demonstrated that *Foxj1* is a direct target of TAp73, supporting a model in which p73 acts as a regulator of multiciliogenesis through direct and indirect regulation of key genes (Marshall et al., 2016; Nemajerova et al., 2016). Altogether, it was confirmed that TAp73 is at the center of the regulatory network of multiciliogenesis and that it is necessary and sufficient to activate the multiciliogenesis program in tracheal cells, and required in MCC from brain ventricles, oviduct, middle ear, sinus mucosa and flagella of sperm in the testis (Gonzalez-Cano et al., 2016; Marshall et al., 2016; Nemajerova et al., 2016; Fujitani et al., 2017). However, it is plausible that p73 acts, at least in brain, at more of one stage of the MCC generation process.

## *Trp73* is Required for Multiciliogenesis of ECS

After birth, the initial steps of ECs differentiation imply a massive production of Basal Bodies (BBs) and their migration and docking to the apical membrane where they will nucleate motile cilia (Spassky and Meunier, 2017). Centriolar amplification requires the initial aggregation of immature centrioles into procentriole organizers, named deuterosomes. At the last stages, the centrioles will disengage from the deuterosome platforms, migrate to the apical membrane and the deuterosomes will disappear (Spassky and Meunier, 2017). In the absence of p73, BBs aggregate into deuterosomes, but these structures do not completely disappear and are detected in ECs from young (P15) and adult (P30) p73KO mice, suggesting the process is halted or defective (Gonzalez-Cano et al., 2016). Following their amplification, centrioles migrate to the apical surface where they become BBs (Figure 1A). p73KO ECs, as well as TAp73KO, have higher number of BBs scattered and deep into the cytoplasm, confirming the required role of TAp73 in centriolar amplification and docking in ECs (Figure 1B) (Fuertes-Alvarez et al., 2018). These defects might be due to TAp73 regulation of the MCC factors, *Foxj1* and *Myb*, both downstream of TAp73 (Marshall et al., 2016; Nemajerova et al., 2016), but also due to defects in actin-cytoskeleton dynamics (Fuertes-Alvarez et al., 2018).

ECs motile cilia directly emerge from the apical membrane in mature tissues (Figures 1A,B) following the extracellular ciliogenesis pathway (Mirzadeh et al., 2008). Thus, defects in BB docking could result in defective axoneme elongation. As expected, p73KO-ECs with total lack of p73 have severe ciliary defects, with many cells lacking ciliary axoneme and others displaying disorganized cilia of different lengths (Gonzalez-Cano et al., 2016) (Figures 1B–D). It is noteworthy, that cells that only lack the TAp73 isoform (TAp73KO) had defective BB docking and showed ciliary defects like disorganized cilia with a “disheveled” appearance (Fuertes-Alvarez et al., 2018). This suggested defects in BB organization and phenocopied the *Celsr2/3* mutant mice where the unidirectional orientation of the BB of their motile cilia, named rotational Planar Cell Polarity (rPCP), was not established (Tissir et al., 2010). However, most of the TAp73KO-ECs, although not all, have ciliary axoneme, displaying a milder ciliary-phenotype than p73KO-ECs (Fuertes-Alvarez et al., 2018) (Figure 1D). Nevertheless, additional studies are required to determine the extent of the effect of these alterations in ciliary function. On the other hand, and consistent with the lack of DNp73 expression in ECs (Tissir et al., 2009), DNp73KO-ECs do not display any cilia defects (Fuertes-Alvarez et al., 2018) indicating that, in the presence of TAp73, DNp73 is not necessary for ciliogenesis regulation. These data suggest that compensatory and redundant ciliary programs are induced in the absence of TAp73 when DNp73 is present, but not with total p73 deficiency. Thus, one might speculate that, in this scenario, compensatory DNp73 upregulation could induce, directly or indirectly, key ciliogenesis regulators, downstream of TAp73 function, suggesting a requirement for DNp73.

Centriolar migration, docking and spacing require the organization of the actin cytoskeleton. BBs are transported, *via* an actin-myosin-based mechanism, to the apical surface (Boisvieux-Ulrich et al., 1990; Meunier and Azimzadeh, 2016). *Trp73* deficiency profoundly affects actin cytoskeleton in ECs, resulting in lack of the apical and subapical actin networks and scattered disposition of the BBs in the apical surface (Figure 1B). Subapical actin networks are required for BB spacing, establishment of global coordination of cilia polarity and metachronal synchrony (Werner et al., 2011). Our group have recently discovered that TAp73, in addition of its regulation of the ciliary programs, can regulate the activity and localization of the actin-binding protein non-muscle myosin II (NMII) (Vicente-Manzanares et al., 2009; Kishimoto and Sawamoto, 2012), via the transcriptional activation of its regulatory kinase gene: the myosin light polypeptide kinase *MLCK* (Fuertes-Alvarez et al., 2018). Thus, TAp73 regulation of *MLCK* directly links p73 with actin microfilament dynamics, ciliogenesis and the mechanisms that orchestrate cellular polarity.

## p73 Regulation of Planar Cell Polarity

Cilia maturation during EC differentiation requires the polarization and organization of the BBs for the cilia to beat in a synchronized manner and create directional fluid flow. This polarization, orthogonal to the apico-basal axis, is known as Planar Cell Polarity (PCP) and is acquired by ECs during their differentiation in a simultaneous process to multiciliogenesis (Ohata and Alvarez-Buylla, 2016) (Figure 1A). ECs display two types of PCP: the asymmetric localization of the cilia cluster at the anterior apical surface following the cerebral spinal fluid (CSF) flow, named translational polarity (tPCP), and rotational polarity (rPCP) (Mirzadeh et al., 2010). Combined staining of the centriolar satellite protein FOP (FGFR1 Oncogene Partner) and γ-tubulin is a frequently used indicator to delineate BB orientation and rPCP (Boutin et al., 2014) (Figure 1D). Unlike p73KO or TAp73KO mice, tumor-prone p53 KO mice do not present ependymal ciliary PCP defects, indicating that regulation of ciliogenesis and PCP is a p73-specific function (Figure 1D).

tPCP is first observed in RGCs as early as E16, when primary cilia become asymmetrically displaced in their apical surface (Mirzadeh et al., 2010). *Trp73* is necessary for the efficient establishment of tPCP in RGC and in immature ECs, and in the absence of p73, or TAp73, tPCP fails to be established (Gonzalez-Cano et al., 2016; Fujitani et al., 2017; Fuertes-Alvarez et al., 2018). Actin microfilament remodeling and NMII activity are essential to establish tPCP (Hirota et al., 2010). NMII is activated (p-MLC) by phosphorylation of its associated regulatory light chain by MLCK and ROCK kinases, but only MLCK activation regulates tPCP (Hirota et al., 2010). NMII activation and its polarized membrane localization in puncta at membrane junctions correlates with anterior migration of the crescent-shaped BB clusters and tPCP establishment (Figure 1B). TAp73 regulates tPCP through the modulation of NMII activity and localization, which are impaired in the absence of p73 (Fuertes-Alvarez et al., 2018).

PCP is regulated by asymmetric signaling through core and global regulatory modules. PCP-core module includes Frizzled (Fzd3–6), Van Gogh-like (Vangl1/2), cadherin epidermal growth factor (EGF)-like laminin G-like seven-pass G-type receptor (Celsr1–3), Dishevelled (Dvl1–3), and Prickle (Pk1–4). In ECs, asymmetric localization of the PCP-core complexes at opposite sides of the apical membrane is required for the rotational and translational-PCP (Boutin et al., 2014; Ohata et al., 2014). Adaptor proteins Dvl2, is detected in close association with BBs following a polarized distribution, highlighting its role in instructing polarization to the BBs and coupling BBs-associated information with the rest of the cell (Ohata and Alvarez-Buylla, 2016; Fuertes-Alvarez et al., 2018) (Figure 1B). Molecular complexes formed by Dvl and Daple at the base of the BBs may regulate ependymal PCP by activating effectors like aPKC, Rac1, and RhoA, leading to reorganization of the actin cytoskeleton (Ohata et al., 2014). Rac1 is localized on top of the BBs, at the base of the axoneme (Fuertes-Alvarez et al., 2018), which probably corresponds to the ciliary pocket, a proposed interface with the actin cytoskeleton and a platform for vesicle trafficking (Figure 1B) (Molla-Herman et al., 2010).

In ECs, mutations of the PCP-core genes *Celsr2/3, Vangl2* and *Dvl2* affect BB-docking and rPCP (Guirao et al., 2010; Hirota et al., 2010; Tissir et al., 2010), while alterations in *Celsr1, Fzd3* and *Vangl2* impair tPCP (Boutin et al., 2014). In this context, ablation of *Trp73* or TAp73, results in the absence of PCP-asymmetric complexes formation (Figure 1B) and lack of both, translational and rotational PCP (Figure 1D), suggesting that p73 might regulates early up-stream events of PCP establishment. Microtubules crosstalk with PCP at two stages: at the initial polarization establishment and as the downstream-effector of PCP (Vladar et al., 2012; Werner and Mitchell, 2012; Matis et al., 2014; Carvajal-Gonzalez et al., 2016b). Polarized microtubules (MTs) lay the tracks for PCP-core polarized trafficking and are required for rPCP establishment prior to PCP-core-module dissymmetry (Shimada et al., 2006; Harumoto et al., 2010).

In Drosophila the global module formed by the protocadherin Fat and its ligand Dachsous (Fat/Ds), acts by controlling the alignment and asymmetry of MT dynamics, promoting an initial polarization of the apical junctional MT which induces the asymmetric distribution of PCP-core complexes (Harumoto et al., 2010; Matis et al., 2014). As described in MTEC (Dau et al., 2016; Ohata and Alvarez-Buylla, 2016), in ECs Fat4 is located at the base of the cilia associated with the BBs (Figure 1B) (Fuertes-Alvarez et al., 2018). p73 deficiency blunts the formation of polarized MT-anchoring points at cell junctions, marked by EB3 staining (Boutin et al., 2014; Takagishi et al., 2017) (Figure 1B), suggesting that MT-dynamics impairment is at the root of the defect in p73-deficient cells (Boutin et al., 2014; Takagishi et al., 2017).

## Causal Relationship Between of PCP and Ciliogenesis and p73 Regulation

The causal relationship between ciliogenesis and PCP signaling is not fully deciphered (Wallingford, 2010). In ECs, PCP coordinately orients the cilia within cells and across the tissue and disruption in PCP establishment results in dysfunctions of ependymal cilia, implicating PCP signaling upstream of ciliogenesis (Boutin et al., 2014). However, elimination of motile cilia by conditional ablation of the ciliogenesis gene *Kif3a* (Kozminski et al., 1995) disrupted rotational orientation, but not tPCP (Mirzadeh et al., 2010). Moreover, in airway epithelia PCP depends on multiciliated cell differentiation and ciliogenesis (Vladar et al., 2016), indicating that cilia may regulate PCP.

Another point of discrepancy is whether PCP-proteins play a role orienting ciliary positioning or if there is also a crosstalk with ciliogenesis. Compiled data on *Vangl* mutants in mice and zebrafish strongly argue for a role of PCP-signaling upstream of ciliary positioning, but not in ciliogenesis *per se* [reviewed in (Carvajal-Gonzalez et al., 2016a)]. However, several PCP-mutants, like *Celsr2/3*-mutant mice, display ciliogenesis defects, linking the PCP-core factors to the ciliogenesis process (Tissir et al., 2010). New studies in *Drosophila* in non-ciliated epithelium establish that PCP-core signaling is an evolutionary conserved module, that acts upstream of centriole positioning independently of ciliogenesis (Carvajal-Gonzalez et al., 2016b).

The results regarding *Trp73* function in ciliogenesis and PCP situates this gene at the epicenter of this conundrum. TAp73 is a master regulator of ciliogenesis and *Trp73* total loss results in dramatic ciliary defects in oviduct, middle ear, respiratory tract and ECs, among others (Gonzalez-Cano et al., 2016; Marshall et al., 2016; Nemajerova et al., 2016; Fujitani et al., 2017). *Trp73* also regulates PCP through TAp73-regulation of actin and MT cytoskeleton dynamics (Fuertes-Alvarez et al., 2018). Moreover, TAp73 is necessary and sufficient for PCP-core protein membrane localization in a non-ciliated cellular model, indicating that p73-regulation of PCP is independent of its function in multiciliogenesis (Figure 1A). Interestingly, lack of TAp73 in ECs results in defective actin and MT networks and loss of PCP, but has mild effect on axonemal growth (Fuertes-Alvarez et al., 2018) (Figure 1D). Therefore, we propose that the *Trp73-*mutant mice (p73KO, TAp73KO and DNp73KO) represent a model that uncouples ciliogenesis from PCP.

## *Trp73* Mutant Mice as a Model of Human Congenital Hydrocephalus

p73KO mice exhibit congenital hydrocephalus that, in some cases, can progress to a severe communicating form (Yang et al., 2000). Considering the profound ependymal defects observed in the absence of p73, it feasible to hypothesize that the constitutive and inducible *Trp73* mutant mice could be useful models to increase our understanding of certain ciliopathies, in particular the ones that are accompanied by hydrocephalus (Fujitani et al., 2017). Hydrocephalus is a condition that can be caused by a variety of factors; thus, it is possible that the lack of total *Trp73* generates more that one of these situations. As proposed by Pozniak et al. (2002), cellular loss due to p53-induced apoptosis in the absence of DNp73 is probably one of the reasons of the hydrocephalus observed in p73KO mice. However, it is unlikely that this would be the only cause, since the double mutant p53KO/p73KO (DKO) mice, which lacks both apoptotic inducers, p53 and TAp73, developed ventriculomegaly shortly after birth (Gonzalez-Cano et al., 2016). Thus, other p73-dependent functions, different from DNp73-cell survival, should also be implicated in the development of p73KO-hydrocephalus. Both mutant mice, p73KO and DKO, have defects in PCP establishment and an abnormal ependymal layer with ECs that does not establish appropriate cell–cell contacts (Gonzalez-Cano et al., 2016). Interestingly, previous work had reported that loss of cell adhesion due to defective Non muscle myosin II, a tPCP regulator, can cause hydrocephalus (Ma et al., 2007). Other groups have shown that defects in tPCP in radial glia progenitors and in tissue polarity result in hydrocephalus (Hirota et al., 2010; Boutin et al., 2014; Takagishi et al., 2017). In that regard, our group and others have demonstrated that TAp73 is required for the establishment of tPCP (Gonzalez-Cano et al., 2016; Fujitani et al., 2017; Fuertes-Alvarez et al., 2018), a process that begins during late embryonic stages and determines primary cilia displacement and future positioning of mature ependymal cilia-tufts. Thus, it is feasible to hypothesize that embryonic p73-function in the establishment of tPCP in RGCs is crucial for hydrocephalus. In agreement with this, Fujitani and colleagues, using a conditional mutant, found that disruption of *Trp73* (both TA and DNp73) during early postnatal ependymal cells development (P1-P5) did not cause hydrocephalus and proposed that embryonic primary ciliogenesis may be regulated by p73 and this would be critical for hydrocephalus (Fujitani et al., 2017). Nevertheless, it is important to keep in mind that TAp73 central and required role for multiciliogenesis and *Foxj1* expression may also be fundamental for hydrocephalus (Marshall et al., 2016; Nemajerova et al., 2016), since disruption of *Foxj1* expression in ECs induces EC transformation, ventricular breakdown, and hydrocephalus (Abdi et al., 2018).

Congenital hydrocephalus (CH) is a major cause of childhood morbidity and mortality, affecting 4.65 per 10,000 births (Shaheen et al., 2017). Despite its prevalence, the etiology of this abnormal accumulation of CSF in the brain remains largely uncharacterized. Although it is known that the genetic component plays an important role in the development of the disease, only few genes have been identified as the primary cause of CH including: *L1CAM, AP1S2, MPDZ, CCDC88C, EML1,* and *WDR81* (Tully and Dobyns, 2014; Kousi and Katsanis, 2016; Shaheen et al., 2017). Recently, four neurogenesis-associated genes: *TRIM71, SMARCC1, PTCH1,* and *SHH*, have been added to this list, attracting attention to the fact that some communicating forms of CH may be related to impaired neurogenesis (Furey et al., 2018). Under this scenario, p73 mutant mice could also be a relevant model, since p73 deficient mice display a defective neurogenic capacity (Gonzalez-Cano et al., 2010, 2016).

Hydrocephalus can also be linked to primary ciliary dyskinesia (PCD), an autosomal recessive disorder of cilia ultrastructure and function. Although this clinical manifestation of PCD is not frequently diagnosed in humans, it is highly prevalent in mice and therefore, mouse models could help to understand the differences in susceptibility to this pathology (Norris and Grimes, 2012; Lee, 2013). Mutations in five genes (*DNAH5, DNAH11, DNAI1, CCDC39, CCDC40*) encoding proteins necessary for motile cilia function are the most common in PCD (Knowles et al., 2016), some of which were identified as direct TAp73-transcriptional targets by Nemajerova et al. (2016) in MTEC cultures.

**Figure 2:**
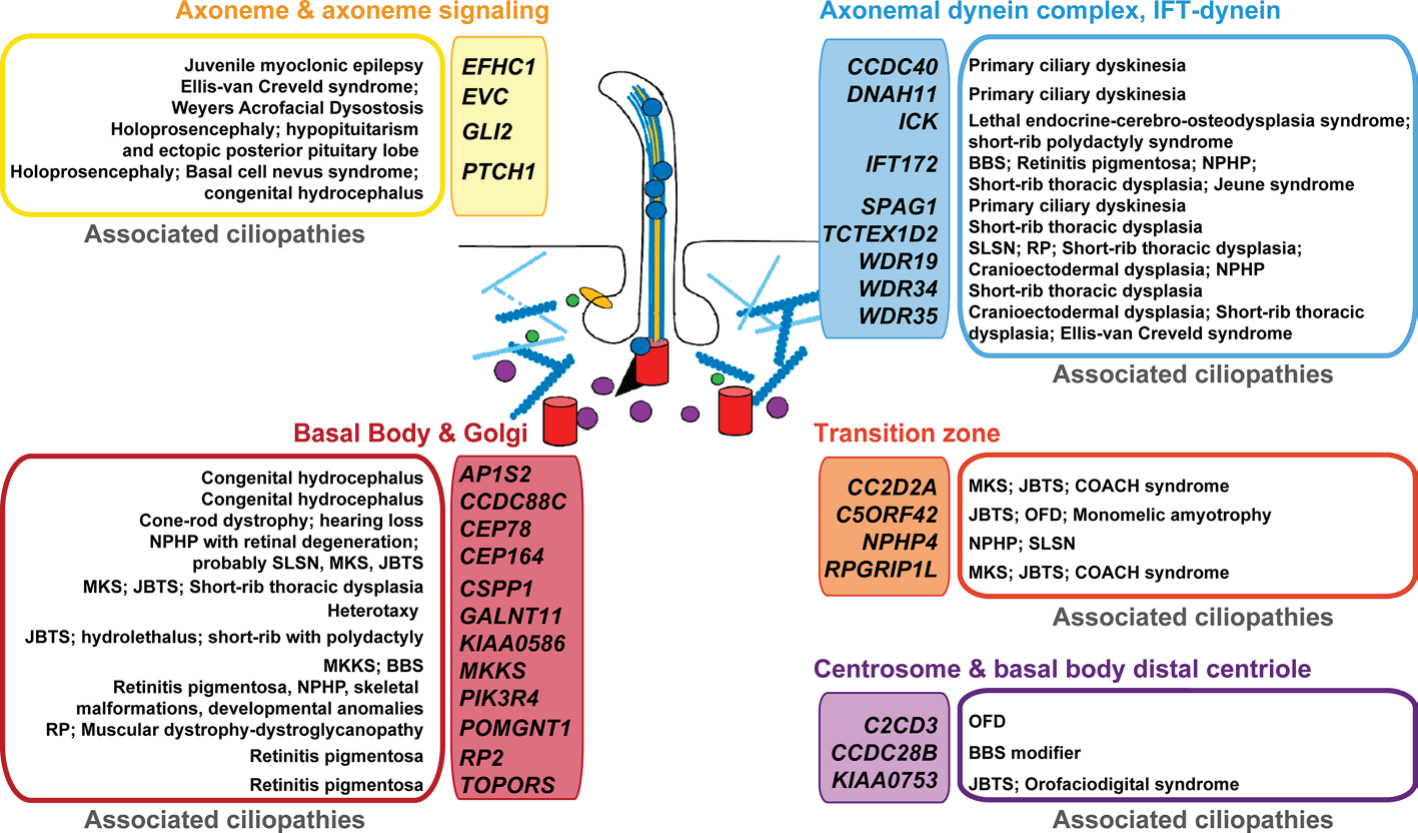
Putative p73 transcriptional target genes and their association with ciliopathies. Human genes with established or likely roles in cilia were compared with genes that were differentially expressed in TAp73β-expressing Saos-2 cells and which contain TAp73 binding sites within 25 kb of the transcription start site (as identified by ChIP-seq analysis, Koeppel et al., 2011). Colors are used to differentiate the protein localization or functional categorization of the candidate p73 target- “ciliopathy-genes”. The established ciliopathies linked to these genes (Reiter and Leroux, 2017) are grouped and shown beside. BBS: Bardet-Biedl syndrome; JBTS: Joubert syndrome; MKS: Meckel syndrome; MKKS: McKusick-Kaufman syndrome; NPHP: nephronophthisis; OFD: Orofaciodigital syndrome; RP: retinitis pigmentosa; SLSN: Senior-Loken syndrome.

Using a compiled list of human cilium-related genes linked to ciliopathies (Reiter and Leroux, 2017), we performed a cross-analysis with potential p73 target genes (Figure 2) identified in TAp73β-expressing Saos-2 cells (Koeppel et al., 2011). Supporting our hypothesis, the comparison uncovered some of the above mentioned genes as putative p73 transcriptional targets. These include genes encoding the adaptor protein complex 1- AP1S2, the primary receptor for Hedgehog ligands-PTCH1, the dynein axonemal heavy chain DNAH11, or the trafficking component SNX27. Of particular interest is the emergence of *CCDC88C* (encoding DAPLE) as candidate target for p73 regulation, since Daple-KO mice not only have hydrocephalus but also show defects in cell polarity and microtubule dynamics (Takagishi et al., 2017).

Considering the recent body of work regarding p73 role in ciliogenesis and the growing interest in ciliopathies, p73 mutant mice could become a prominent model system for studying the molecular genetics of cilia-associated disorders and, in particular, the possible crosstalk with PCP deregulation in the pathogenesis of these diseases.

## Data Availability

Publicly available datasets were analyzed in this study. This data can be found here: https://academic.oup.com/nar/article/39/14/6069/1369723#supplementary-data.

## Author Contributions

MMM and MCM took the lead in writing and editing the review. JV-F and SF-A obtained data, crafted figures and drafted the work and LM-A performed data analysis. All authors provided critical feedback and revised the manuscript.

### Conflict of Interest Statement

The authors declare that the research was conducted in the absence of any commercial or financial relationships that could be construed as a potential conflict of interest.
